# Lifestyle factors and primary glioma and meningioma tumours in the Million Women Study cohort

**DOI:** 10.1038/sj.bjc.6604445

**Published:** 2008-06-17

**Authors:** V S Benson, K Pirie, J Green, D Casabonne, V Beral

**Affiliations:** 1 Cancer Epidemiology Unit, Nuffield Department of Clinical Medicine, University of Oxford, Oxford, UK

**Keywords:** body mass index, glioma, height, meningioma, women

## Abstract

Previous studies have reported inconsistent results on the effect of anthropometric and lifestyle factors on the risk of developing glioma or meningioma tumours. A prospective cohort of 1.3 million middle-aged women was used to examine these relationships. During 7.7 million women-years of follow-up, a total of 1563 women were diagnosed with a primary incident central nervous system tumour: 646 tumours were classified as glioma and 390 as meningioma. Our results show that height is related to the incidence of all central nervous system tumours with a risk of about 20% per 10 cm increase in height (relative risk=1.19, 95% CI=1.10–1.30 per 10 cm increase in height, *P*<0.001): the risks did not differ significantly between specified glioma and meningioma. Body mass index (BMI) was also related to central nervous system tumour incidence, with a risk of about 20% per 10 kg m^−2^ increase in BMI (relative risk=1.17, 95% CI=1.03–1.34 per 10 kg m^−2^ increase in BMI, *P*=0.02). Smoking status, alcohol intake, socioeconomic level, parity, age at first birth, and oral contraceptive use were not associated with the risk of glioma or meningioma tumours. In conclusion, for women in the United Kingdom, the incidence of glioma or meningioma tumours increases with increasing height and increasing BMI.

Primary brain and central nervous system cancers are relatively rare and represent approximately 2% of all cancers diagnosed in the United Kingdom. However, due to a poor prognosis, they are responsible for 7% of the years of life lost from cancer before the age of 70 years (Cancer Research UK, 2007). Very little is known about the aetiology of central nervous system tumours, but environmental factors are thought to play a role (McKinney, 2004; Connelly and Malkin, 2007).

The two most common types of central nervous system tumour are glioma and meningioma (Claus *et al*, 2005). Gliomas arise from glial cells, are found predominantly in the brain and to a lesser extent in the spinal cord or other parts of the central nervous system, and represent more than 70% of all brain tumours (Ohgaki and Kleihues, 2005). Gliomas are typically histologically malignant, can be either slow or fast growing, and are more frequently diagnosed in men than in women. Meningiomas arise from the arachnoidal cells of the leptomeninges (the pia mater and arachnoid mater of the meninges) (Sanson and Cornu, 2000), are also more frequently found in the brain than elsewhere in the central nervous system, and represent more than 20% of all brain tumours (Longstreth *et al*, 1993). Meningiomas are typically benign (>90%) and slow growing. The risk of meningioma increases with age (Sanson and Cornu, 2000), and they are more frequently diagnosed in women (Sanson and Cornu, 2000; Perry *et al*, 2007).

There are few well-established risk factors for glioma and meningioma tumours among adults; while exposure to ionizing radiation and rare inherited genetic conditions such as neurofibromatosis (Martuza *et al*, 1988) are known to increase risk, these factors explain only a small fraction of brain tumours reported (McKinney, 2004). Several environmental factors (including parity, smoking, mobile phone use, head trauma, and occupational exposure) have been postulated as linked to tumour development, mostly in case–control studies, but the evidence is generally weak or inconsistent (Lambe *et al*, 1997; Inskip *et al*, 1998; Hu *et al*, 1999; Navas-Acién *et al*, 2002; Hepworth *et al*, 2006; Hardell *et al*, 2007). As central nervous system tumours are relatively uncommon, studies of these tumours are limited by the small number of cases, especially in cohort studies.

We report here results from analyses of the relationship between anthropometric and lifestyle factors and the incidence of all central nervous system tumours and of specified gliomas and meningiomas in a large prospective cohort.

## Materials and methods

### Study population

During May 1996 to March 2001, 1.3 million middle-aged women were recruited into the Million Women Study cohort, completing a recruitment questionnaire about reproductive factors, sociodemographic factors, and other personal characteristics. Full details of the study design and methods are described elsewhere (Beral, 1999) and the questionnaire can be viewed at http://www.millionwomenstudy.org. In brief, the Million Women Study was designed as a prospective investigation into women's health, with a particular emphasis on breast cancer and exogenous hormone use. All study participants have been flagged on the National Health Service (NHS) central registers, so that tumour registrations (benign and malignant) and deaths are routinely notified to the study investigators. This information includes the date of each such event and codes the tumour site and morphology using the 10th revision of the International Classification of Diseases (ICD-10) (World Health Organization, 1992) and the 3rd Edition of Morphology and Neoplasms (Fritz *et al*, 2000).

### Data collection

Incident central nervous system tumours were included from the following sites: ICD-10 C70, C71, C72.0, C75.1–3, D32, D33, D35.2–4, D42, D43, and D44.3–5. Incident cases of glioma, morphology codes ICD-O 9380–9481, and meningioma, morphology codes ICD-O 9530–9539, were identified within these sites.

### Statistical analysis

Women diagnosed before recruitment with any malignant tumour (other than non-melanoma skin cancer (C44)) or a benign brain or central nervous system tumour were excluded from the analysis. In addition, we excluded women who reported having the inherited disorder neurofibromatosis (Q85.0), a disorder of the nervous system associated with a high risk of neurological tumours. Eligible women contributed person years from the date of recruitment until the date of registration with the tumour of interest, date of death, or end of follow-up, whichever was the earliest. In addition, women diagnosed with any cancer other than the cancer of interest (except non-melanoma skin cancer) during the follow-up period were censored at the date of diagnosis of that cancer. The end of follow-up for cancer incidence was 31 December 2005 for all registries except Thames, North West (Mersey), and Northern and Yorkshire regions, where follow-up was to 31 December 2004, the West Midlands where it was 30 June 2005 and Scotland where it was 31 December 1999, as these regions are complete only to these dates. Follow-up was complete for over 99% of the cohort population.

We considered all central nervous system tumours and each of the specified tumour types (glioma and meningioma) as separate end points in a Cox proportional hazards model with attained age as the underlying time variable. We stratified analyses by broad geographical region (10 regions corresponding to the areas covered by the cancer registries), and we made adjustments for height (<160, 160–164.9, ⩾165 cm), body mass index (BMI) (<25, 25–29.9, ⩾30 kg m^−2^), socioeconomic status based on deprivation index (Townsend *et al*, 1988) (divided into thirds), smoking status (never, past, current smoker), alcohol intake (never, <1, ⩾1 U per day), strenuous exercise (<1, 1, ⩾2 times per week), age at first birth (<20, 20–24, ⩾25 years old), parity (full-term pregnancies) (0, 1–2, ⩾3), and oral contraceptive use (never, <5 years, ⩾5 years), where appropriate. We assigned women with missing values for any of the adjustment variables to a separate category for that variable. All exposure data were self-reported at recruitment.

Analyses adjusted only by age at recruitment and region were conducted initially to estimate the relationship of each variable in turn to the risk of all central nervous system tumours, glioma, and meningioma. Subsequently, analyses were mutually adjusted for all exposure variables to allow for possible confounding between variables, as appropriate. The significance of categorical variables was assessed using likelihood ratio tests, with the heterogeneity *P*-values reported in the results. Tests for linear trend were obtained by using the mid point values for each category and treating this scored variable as continuous. These relative risks were compared using a *χ*^2^ test for heterogeneity to see whether the effect of each exposure variable varied between tumour types.

## Results

In total, 1 249 670 women aged between 50 and 65 years were eligible for analysis, with an average age at recruitment of 55.9 years. A total of 1563 incident primary central nervous system tumours were diagnosed after an average of 6.2 years follow-up (7 740 300 woman-years). Of these, 646 tumours were classified as glioma (98% as malignant) and 390 as meningioma (95% as benign). Table 1 shows the baseline characteristics of the study population and of women diagnosed with any central nervous system tumour, with glioma, or with meningioma.

**Figure 1:**
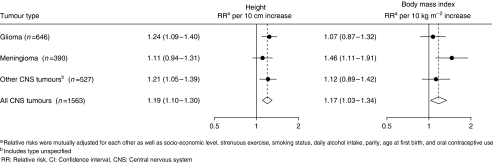
Relative risks for a 10 cm increase in height and 10 kg m^−2^ increase in body mass index.

Table 2 shows relative risks for the incidence of all central nervous system tumours and specified glioma and meningioma, by height, BMI, strenuous exercise, socioeconomic level, smoking status, alcohol intake, parity, age at first birth, and use of oral contraceptives. Adjusted and unadjusted analyses gave very similar results for all tumours and for each specified tumour type. The relative risk for all central nervous system tumours increased with increasing height, as did the relative risks of both glioma and meningioma. The association of height and tumour risk was significant for all central nervous system tumours (*P* for heterogeneity <0.001) and modestly significant for glioma (*P* for heterogeneity=0.02). For meningioma, although the association was not statistically significant, the relative risks were similar to those for all central nervous system tumours and glioma (Table 2). Figure 1 shows the relative risk for a 10 cm increase in height and tumour risk; relative risk for the incidence of all central nervous system tumours increased by a factor of 1.19 per 10 cm increase in height. For glioma, meningioma, and other central nervous system tumours, the equivalent increase in risk was by factors of 1.24, 1.11, and 1.21, respectively, and there was no significant difference between these relative risks (*P* for heterogeneity=0.6).

Body mass index was also related to the incidence of all central nervous system tumours, as well as to the incidence of meningioma, and to a lesser extent, glioma (Table 2). For all tumours and for meningioma, the association was statistically significant (*P* for heterogeneity=0.02, 0.03, respectively), with obese women (BMI⩾30 kg m^−2^) having a relative risk of 1.21 (95% CI=1.04–1.39) and 1.40 (95% CI=1.08–1.87), respectively, when compared to women with a BMI <25 kg m^−2^. For glioma, obese women had a non-significant relative risk of 1.07 (95% CI=0.84–1.34). Figure 1 shows that for all central nervous system tumours, the relative risk per 10 kg m^−2^ increase in BMI increases by a factor of 1.17; and by 1.07, 1.46, and 1.12 for the incidence of glioma, meningioma, and other central nervous system tumours, respectively. Again, no significant difference between these relative risks for glioma, meningioma, and other central nervous system tumours was observed (*P* for heterogeneity=0.2).

Strenuous exercise was also related to the incidence of all central nervous system tumours, glioma, and meningioma, with a slightly lower risk in women who reported strenuous exercise once per week or more often compared to women who exercised less than once per week or never. Again, there was no significant difference between the relative risks for glioma, meningioma, and other central nervous system tumours, when comparing less than weekly to at least weekly strenuous exercise (*P* for heterogeneity=0.2).

Socioeconomic level, daily alcohol intake, smoking status, number of full-term pregnancies, age at first birth, and oral contraceptive use were not associated with the incidence of all central nervous system tumours, glioma, or meningioma.

The main analyses reported here focused on morphologically specified glioma and meningioma tumours. Of the 527 ‘other central nervous system tumours’ (as shown in Figure 1), 148 were sited in the brain and 52 in the meninges, but all had non-specific morphology codes. As all but 9 of the 646 specified gliomas were sited in the brain (the remainder were in the spinal cord) and all but 4 meningiomas were sited in the meninges (the remainder were in the brain), it is reasonable to assume that the great majority of morphologically unspecified tumours of the brain are likely to be gliomas; and of the meninges, meningioma. Sensitivity analyses showed that for a 10 cm increase in height, the relative risks were very similar for glioma (*n*=646) and for all brain tumours (*n*=789) (relative risk=1.24 (95% CI=1.09–1.40) and relative risk=1.20 (95% CI=1.07–1.34), respectively), and for meningioma (*n*=390) and for all meninges tumours (*n*=438) (relative risk=1.11 (95% CI=0.94–1.31) and relative risk=1.17 (95% CI=1.00–1.37), respectively). Similarly, for a 10 kg m^−2^ increase in BMI; the relative risks for glioma and for all brain tumours were 1.07 (95% CI=0.87–1.32) and 1.04 (95% CI=0.86–1.25), respectively, and the relative risks for meningioma and for all meninges tumours were 1.46 (95% CI=1.11–1.91) and 1.54 (95% CI=1.20–1.99), respectively. The remaining central nervous system tumours included 173 tumours of the cranial nerves (150 were of the 8th cranial nerve), 125 of the pituitary gland, 10 of the spinal cord, 6 central nervous system tumours not otherwise specified, and 13 other central nervous system tumours. The number of tumours in these categories are at present too small to allow reliable analysis.

## Discussion

Increasing height and BMI are associated with increasing incidence of all central nervous system tumours, glioma, and meningioma in this large cohort of middle-aged women.

Tumours of the brain and central nervous system are relatively uncommon, and most of the evidence for potential risk factors comes from case–control studies, as there are insufficient cases for reliable estimations in most cohort studies. In addition, as meningiomas are typically benign, their incidence is not reported to cancer registries in some countries; therefore, these tumours are not always included in some studies. Existing evidence on the role of environmental risk factors and the incidence of glioma and meningioma is thus limited.

For tumours of the central nervous system, there is limited and inconsistent evidence about the effects of height (Helseth and Tretli, 1989; Lee *et al*, 1997; Tulinius *et al*, 1997; Gunnell *et al*, 2001). Our results provide strong evidence that height is related to the incidence of central nervous system tumours with a risk of about 20% per 10 cm increase in height, with no significant differences between the effects for specified glioma and meningioma. An association of this magnitude is consistent with that seen between adult height and the incidence of several other common cancers, including those of the breast and colon (Gunnell *et al*, 2004; World Cancer Research Fund/American Institute for Cancer Research, 2007). The suggested mechanisms underlying these associations include a simple link between height and cell number; and the relation between rates of childhood growth and levels in childhood and adulthood insulin-like growth factors, which may influence cell proliferation and tumour growth (Gunnell *et al*, 2004).

Three of four previous cohort studies reported an increased risk of the incidence of central nervous system tumours in relation to obesity (Albanes and Taylor, 1990; Møller *et al*, 1994; Tulinius *et al*, 1997), and one reported no association (Oh *et al*, 2005). Evidence from case–control studies is also inconsistent (Bellur *et al*, 1983; Helseth and Tretli, 1989; Schneider *et al*, 2005). This present detailed analysis shows an increasing risk with increasing BMI and central nervous system tumours (including specified glioma and meningioma). Obesity may be related to cancer risk through several possible mechanisms, including increased inflammatory response, decreased insulin sensitivity and, particularly in women, through increases in circulating oestrogen levels (Reeves *et al*, 2007; World Cancer Research Fund/American Institute for Cancer Research, 2007). More evidence is needed on the interrelated exposures of body size and both endogenous and exogenous hormones in relation to brain cancer to explore possible hormonal mechanisms (Claus *et al*, 2005).

We are not aware of any previous studies with evidence of an association between physical activity and central nervous system tumours. The main difference appeared to be between little or no strenuous activity and some, with little suggestion of any association with the amount of exercise. It is possible that the higher risk seen with little or no strenuous exercise is because tumour symptoms such as headache may have prevented strenuous exercise. Exclusion of the first 2 years of follow-up, which should reduce this possible bias, did not materially affect the relative risks in relation to strenuous exercise: for all central nervous system tumours, the relative risks for strenuous exercise once per week and two or more times per week compared with little or no strenuous exercise were 0.86 (95% CI=0.75–0.99) and 0.86 (95% CI=0.75–0.98), respectively, and after exclusion of the first 2 years of follow-up, the relative risks were 0.90 (95% CI=0.76–1.06) and 0.82 (95% CI=0.70–0.96), respectively. However, additional evidence is needed before the relevance of these findings can be assessed.

The lack of association between smoking and the incidence of glioma and meningioma is consistent with results from most studies (Mills *et al*, 1989; Hurley, 1996; Zheng *et al*, 2001; Schneider *et al*, 2005), but not all of them (Ryan *et al*, 1992; Efird *et al*, 2004; Navarro Silvera *et al*, 2006a). Furthermore, smoking is not classed as a risk factor for central nervous system tumours by the International Agency for Research on Cancer in a recent evaluation (International Agency for Research on Cancer, 2004). The finding of no association between alcohol intake and the incidence of glioma and meningioma is also supported by several previous studies (Mills *et al*, 1989; Hurley, 1996; Hu *et al*, 1999; Efird *et al*, 2004).

Parity and age at first birth have been associated with glioma incidence in two studies (Lambe *et al*, 1997; Navarro Silvera *et al*, 2006b), but not with meningioma incidence (Lambe *et al*, 1997; Jhawar *et al*, 2003); no association was evident in our study. In addition, as in this study, previous studies have found no association between oral contraceptive use and meningioma or glioma risk (Jhawar *et al*, 2003; Custer *et al*, 2006; Wigertz *et al*, 2006).

The main strength of this study is that it is prospective; therefore, recall bias was not a concern. Complete follow-up is available for over 99% of the participants through the NHS central registers with quality assurance of data. The very large study size allows reliable estimation of relative risks for specific tumour types defined by histology, even for less common tumours. The number of cases in the Million Women Study cohort is greater than the total in all previous cohort studies of glioma or meningioma and similar risk factors.

The main limitation of this study is that exposure data are self-reported. Previous studies investigating the accuracy of self-reported *vs* measured height have shown that self-reported height is a good indicator of measured height (Spencer *et al*, 2002; Brunner, 2007). As with many large epidemiological studies, BMI in our cohort was calculated based on self-reported height and weight, thus is subject to both systematic and random errors. The random component of this error is likely to be small (Whitlock *et al*, 2001). The majority of the women in the study were moderate drinkers (<1 U per day), and so this study cannot examine the effects of heavy drinking (⩾2 U per day) as this group was represented by only 5% of the population.

Our findings indicate that increasing height and increasing BMI increase the incidence of all central nervous system tumours, and of both glioma and meningioma tumours.

## Figures and Tables

**Table 1:** Baseline characteristics of the study population according to the population at risk and tumour incidence

**Characteristic**	**Population at risk**	**All incident central nervous system tumours**	**Incident glioma**	**Incident meningioma**
Number of women	1 249 670	1563	646	390
Mean age at entry (s.d.)	55.9 (4.5)	55.9 (4.5)	57.0 (4.5)	56.4 (4.7)
Upper third of socioeconomic group (*n*, %)	414 816 (33.4)	527 (33.9)	214 (33.2)	134 (34.5)
Mean height, cm (s.d.)	162 (6.7)	163 (6.9)	163 (6.8)	162 (7.0)
Mean body mass index, kg m^−2^ (s.d.)	26.2 (4.7)	26.4 (4.7)	26.2 (4.5)	26.8 (4.7)
Mean parity (s.d.)	2.1 (1.2)	2.2 (1.3)	2.2 (1.2)	2.2 (1.3)
Past use of oral contraceptive use (*n*, %)	742 667 (60.1)	881 (57.0)	339 (53.1)	228 (59.4)
Strenuous physical activity⩾once per week (*n*, %)	469 568 (39.0)	539 (35.8)	223 (35.9)	120 (31.9)
Current smoker (*n*, %)	244 363 (20.8)	256 (17.3)	107 (17.3)	70 (18.8)
Mean alcohol intake, g day^-1^ (s.d.)	6.2 (7.6)	5.9 (7.3)	5.9 (7.4)	6.2 (7.2)

**Table 2:** Relative risk of the incidence of all central nervous system tumours, incident glioma, and incident meningioma, by lifestyle factors

		**All central nervous system tumours (*n*=1563)**			**Glioma (*n*=646)**			**Meningioma (*n*=390)**		
**Variable of interest**	**Population^a^**	**Cases**	**RR^b^**	**RR^c^ (95% CI)**	**Cases**	**RR^b^**	**RR^c^ (95% CI)**	**Cases**	**RR^b^**	**RR^c^ (95% CI)**
*Height (cm)*										
<160	405 831	435	1.00	1.00	177	1.00	1.00	115	1.00	1.00
160–164.9	367 494	459	1.15	1.16 (1.02–1.33)	196	1.21	1.22 (0.99–1.49)	108	1.02	1.05 (0.81–1.37)
165+	456 174	639	1.28	1.31 (1.16–1.48)	262	1.30	1.31 (1.08–1.59)	157	1.19	1.25 (0.98–1.60)
Heterogeneity *p*			<0.001	<0.001		0.02	0.02		0.3	0.2
										
*Body mass index (kg m^−2^)*										
<25	548 846	632	1.00	1.00	259	1.00	1.00	154	1.00	1.00
25–29.9	422 508	543	1.11	1.12 (1.00–1.26)	241	1.20	1.20 (1.01–1.44)	120	1.01	1.01 (0.79–1.29)
30+	212 871	289	1.20	1.21 (1.04–1.39)	106	1.06	1.07 (0.84–1.34)	84	1.42	1.40 (1.08–1.87)
Heterogeneity *P*			0.03	0.02		0.1	0.1		0.03	0.03
										
*Strenuous exercise*										
<1 per week	733 886	966	1.00	1.00	398	1.00	1.00	256	1.00	1.00
1 per week	216 414	249	0.87	0.86 (0.75–0.99)	99	0.85	0.84 (0.67–1.05)	55	0.72	0.73 (0.55–0.98)
2+ per week	253 154	290	0.86	0.86 (0.75–0.98)	124	0.91	0.91 (0.75–1.12)	65	0.72	0.73 (0.56–0.97)
Heterogeneity *P*			0.02	0.02		0.3	0.3		0.01	0.02
										
*Socioeconomic level*										
Upper third	414 816	527	1.00	1.00	214	1.00	1.00	134	1.00	1.00
Middle third	412 630	530	0.99	0.99 (0.88–1.12)	216	1.00	1.01 (0.83–1.22)	125	0.92	0.91 (0.71–1.16)
Lower third	413 041	498	0.98	0.98 (0.87–1.12)	214	1.01	1.04 (0.85–1.27)	129	1.00	0.96 (0.74–1.23)
Heterogeneity *P*			1.0	1.0		0.99	0.9		0.7	0.7
										
*Smoking*										
Never	599 949	819	1.00	1.00	322	1.00	1.00	203	1.00	1.00
Past	332 775	407	0.92	0.91 (0.81–1.03)	189	1.08	1.09 (0.91–1.31)	99	0.89	0.86 (0.67–1.10)
Current	244 363	256	0.84	0.85 (0.74–0.99)	107	0.89	0.91 (0.73–1.15)	70	0.91	0.88 (0.66–1.16)
Heterogeneity *P*			0.04	0.06		0.3	0.3		0.6	0.4
										
*Alcohol intake (g day^−1^)*										
Never	296 765	376	0.98	0.98 (0.86–1.10)	151	0.90	0.90 (0.74–1.09)	92	1.02	0.98 (0.76–1.26)
<1	644 775	834	1.00	1.00	357	1.00	1.00	198	1.00	1.00
1+	298 650	343	0.93	0.95 (0.84–1.08)	136	0.86	0.87 (0.71–1.06)	96	1.07	1.13 (0.88–1.44)
Heterogeneity *P*			0.5	0.7		0.3	0.3		0.9	0.6
										
*Parity*										
0	134 346	166	1.00	1.00	70	1.00	1.00	37	1.00	1.00
1–2	701 479	846	0.97	1.01 (0.80–1.26)	355	0.97	0.92 (0.64–1.32)	215	1.09	1.18 (0.74–1.86)
3+	411 082	549	1.05	1.07 (0.86–1.33)	221	0.99	0.96 (0.68–1.35)	137	1.18	1.22 (0.78–1.89)
Heterogeneity *P*			0.4	0.5		0.96	0.9		0.6	0.7
										
*Age at first birth (years)*										
<20	151 798	184	1.00	1.00	68	1.00	1.00	50	1.00	1.00
20–24	522 365	658	0.97	0.97 (0.82–1.14)	273	1.09	1.08 (0.82–1.41)	169	0.94	0.95 (0.69–1.31)
25+	411 369	532	0.99	0.99 (0.83–1.19)	227	1.14	1.13 (0.84–1.50)	124	0.88	0.89 (0.63–1.27)
Heterogeneity *P*			0.9	0.9		0.6	0.7		0.7	0.8
										
*Oral contraception use duration*										
Never	493 422	664	1.00	1.00	300	1.00	1.00	156	1.00	1.00
<5 years	298 298	365	1.04	1.05 (0.92–1.20)	138	0.89	0.88 (0.72–1.09)	91	1.05	1.06 (0.81–1.38)
5+ years	412 479	480	1.00	1.03 (0.91–1.16)	185	0.88	0.88 (0.72–1.06)	128	1.07	1.10 (0.86–1.40)
Heterogeneity *P*			0.8	0.8		0.3	0.3		0.9	0.8

Abbreviations: RR=relative risk, 95% CI=95% confidence interval.

a Population at risk at the start of the study.

b Relative risks stratified by region with attained age as the underlying time variable.

c Multivariate relative risks mutually adjusting for all other variables in the table.

## Appendix

Million Women Study Collaborators

*Steering Committee*: Joan Austoker, Emily Banks, Valerie Beral, Judith Church, Ruth English, Jane Green, Julietta Patnick, Richard Peto, Gillian Reeves, Martin Vessey, and Matthew Wallis.

*Million Women Study Coordinating Centre Staff*: Simon Abbott, Krys Baker, Angela Balkwill, Emily Banks, Ruanne Barnabas, Vicky Benson, Valerie Beral, Judith Black, Anna Brown, Diana Bull, Rebecca Cameron, Karen Canfell, Delphine Casabonne, Barbara Crossley, Dave Ewart, Sarah Ewart, Lee Fletcher, Toral Gathani, Laura Gerrard, Adrian Goodill, Jane Green, Isobel Green, Elizabeth Hilton, Joy Hooley, Sau Wan Kan, Carol Keene, Ruth Keogh, Nicky Langston, Bette Liu, Sarit Nehushtan, Lynn Pank, Kirstin Pirie, Gillian Reeves, Andrew Roddam, Emma Sherman, Moya Simmonds, Elizabeth Spencer, Richard Stevens, Helena Strange, Siân Sweetland, Alison Timadjer, Sarah Tipper, Joanna Watson, and Stephen Williams.

*Collaborating UK NHS Breast Screening Centres*: Avon, Aylesbury, Barnsley, Basingstoke, Bedfordshire and Hertfordshire, Cambridge and Huntingdon, Chelmsford and Colchester, Chester, Cornwall, Crewe, Cumbria, Doncaster, Dorset, East Berkshire, East Cheshire, East Devon, East of Scotland, East Suffolk, East Sussex, Gateshead, Gloucestershire, Great Yarmouth, Hereford and Worcester, Kent, Kings Lynn, Leicestershire, Liverpool, Manchester, Milton Keynes, Newcastle, North Birmingham, North East Scotland, North Lancashire, North Middlesex, North Nottingham, North of Scotland, North Tees, North Yorkshire, Nottingham, Oxford, Portsmouth, Rotherham, Sheffield, Shropshire, Somerset, South Birmingham, South East Scotland, South East Staffordshire, South Derbyshire, South Essex, South Lancashire, South West Scotland, Surrey, Warrington Halton St Helens and Knowsley, Warwickshire Solihull and Coventry, West Berkshire, West Devon, West London, West Suffolk, West Sussex, Wiltshire, Winchester, Wirral, Wycombe.

## References

[bib1] Albanes D, Taylor PR (1990) International differences in body height and weight and their relationship to cancer incidence. Nutr Cancer 14: 69–77 2367236 10.1080/01635589009514078

[bib2] Bellur SN, Chandra V, Anderson RJ (1983) Association of meningiomas with obesity. Ann Neurol 13: 346–347 6847154 10.1002/ana.410130329

[bib3] Beral V (1999) The Million Women Study: design and characteristics of the study population. Breast Cancer Res 1: 73–80 11056681 10.1186/bcr16 PMC13913

[bib4] Brunner H (2007) Validity of self-reported height and weight in women of reproductive age. Matern Child Health J 11: 137–144 17066316 10.1007/s10995-006-0157-0

[bib5] Cancer Research UK (2007) Brain cancer: UK brain and central nervous system cancer incidence. http://info.cancerresearchuk.org/cancerstats/types/brain/incidence/ (accessed 3 April 2008)

[bib6] Claus EB, Bondy ML, Schildkraut JM, Wiemels JL, Wrensch M, Black PM (2005) Epidemiology of intracranial meningioma. Neurosurgery 57: 1088–1094 16331155 10.1227/01.neu.0000188281.91351.b9

[bib7] Connelly JM, Malkin MG (2007) Environmental risk factors for brain tumours. Curr Neurol Neurosci Rep 7: 208–214 17488586 10.1007/s11910-007-0032-4

[bib8] Custer B, Longstreth WT, Phillips LE, Koepsell TD, Van Belle G (2006) Hormonal exposures and the risk of intracranial meningioma in women: a population-based case–control study. BMC Cancer 6. DOI:10.1186/1471-2407-6-152 10.1186/1471-2407-6-152 PMC1524800 16759391

[bib9] Efird JT, Friedman GD, Sidney S, Klatsky A, Habel LA, Udaltsova NV, Van Den Eeden S, Nelson LM (2004) The risk for malignant primary adult-onset glioma in a large, multiethnic, managed-care cohort: cigarette smoking and other lifestyle behaviors. J Neurooncol 68: 57–69 15174522 10.1023/b:neon.0000024746.87666.ed

[bib10] Fritz A, Percy C, Jack A, Shanmugaratham K, Sobin L, Parkin M, Whelan S (eds) (2000) International Classification of Diseases for Oncology, 3rd edn. World Health Organization: Geneva

[bib11] Gunnell D, Okasha M, Davey S, Oliver SE, Sandhu J, Holly JMP (2001) Height, leg length, and cancer risk: a systematic review. Epidemiol Rev 23: 313–342 12192740 10.1093/oxfordjournals.epirev.a000809

[bib12] Gunnell D, Oliver SE, Donovan JL, Peters TJ, Gillatt D, Persad R, Hamdy FC, Meal DE, Holly JMP (2004) Do height-related variations in insulin-like growth factors underlie the associations of stature with chronic diseases? J Clin Endocrinol Metab 81: 213–218 10.1210/jc.2003-030507 14715852

[bib13] Hardell L, Carlberg M, Söderguist F, Mild KH, Morgan LL (2007) Long-term use of cellular phones and brain tumours: Increased risk associated with use for ⩾10 years. Occup Environ Med 64: 626–632 17409179 10.1136/oem.2006.029751 PMC2092574

[bib14] Helseth A, Tretli S (1989) Pre-morbid height and weight as risk factors for development of central nervous system neoplasms. Neuroepidemiology 8: 277–282 2586697 10.1159/000110195

[bib15] Hepworth SJ, Schoemaker MJ, Muir KR, Swerdlow AJ, van Tongeren MJ, McKinney PA (2006) Mobile phone use and risk of glioma in adults: case–control study. BMJ (Clinical research ed) 332: 883–887 10.1136/bmj.38720.687975.55 PMC1440611 16428250

[bib16] Hu J, Little J, Xu T, Zhao X, Guo L, Jia X, Huang G, Bi D, Liu R (1999) Risk factors for meningioma in adults: a case–control study in northeast China. Int J Cancer 83: 299–304 10495419 10.1002/(sici)1097-0215(19991029)83:3<299::aid-ijc2>3.0.co;2-z

[bib17] Hurley SF (1996) Tobacco smoking and alcohol consumption as risk factors for glioma: a case–control study in Melbourne, Australia. J Epidemiol Community Health 50: 442–446 8882229 10.1136/jech.50.4.442 PMC1060316

[bib18] Inskip PD, Mellemkjaer L, Gridley G, Olsen JH (1998) Incidence of intracranial tumors following hospitalization for head injuries (Denmark). Cancer Causes and Control 9: 109–116 9486470 10.1023/a:1008861722901

[bib19] International Agency for Research on Cancer (2004) Tobacco Smoke and Involuntary Smoking. International Agency for Research on Cancer: Lyon, France

[bib20] Jhawar BS, Fuchs CS, Colditz GA, Stampfer MJ (2003) Sex steroid hormone exposures and risk for meningioma. J Neurosurgery 99: 848–853 10.3171/jns.2003.99.5.0848 14609164

[bib21] Lambe M, Coogan P, Baron J (1997) Reproductive factors and the risk of brain tumors: a population-based study in Sweden. Int J Cancer 72: 389–393 9247278 10.1002/(sici)1097-0215(19970729)72:3<389::aid-ijc2>3.0.co;2-l

[bib22] Lee M, Wrensch M, Miike R (1997) Dietary and tobacco risk factors for adult onset glioma in the San Francisco Bay Area (California, USA). Cancer Causes and Control 8: 13–24 9051318 10.1023/a:1018470802969

[bib23] Longstreth J, Dennis LK, McGuire VM, Drangsholt MT, Koepsell TD (1993) Epidemiology of intracranial meningioma. Cancer 72: 639–648 8334619 10.1002/1097-0142(19930801)72:3<639::aid-cncr2820720304>3.0.co;2-p

[bib24] Martuza RL, Seizinger BR, Jacoby LB, Rouleau GA, Gusella JF (1988) The molecular biology of human glial tumors. Trends Neurosci 11: 22–27 2469150 10.1016/0166-2236(88)90045-8

[bib25] McKinney PA (2004) Brain tumours: incidence, survival, and aetiology. Neurology in Practice 75: ii12–ii17 10.1136/jnnp.2004.040741 PMC1765660 15146034

[bib26] Mills PK, Preston-Martin S, Annegers JF, Beeson WL, Phillips RL, Fraser GE (1989) Risk factors for tumors of the brain and cranial meninges in seventh-day adventists. Neuroepidemiology 8: 266–275 2812186 10.1159/000110193

[bib27] Møller H, Mellemgaard A, Lindvig K, Olsen JH (1994) Obesity and cancer risk: a Danish record-linkage study. Eur J Cancer Part A: General Topics 30: 344–350 10.1016/0959-8049(94)90254-2 8204357

[bib28] Navarro Silvera SA, Miller AB, Rohan TE (2006a) Cigarette smoking and risk of glioma: a prospective cohort study. Inter J Cancer 118: 1848–1851 10.1002/ijc.21569 16217772

[bib29] Navarro Silvera SA, Miller AB, Rohan TE (2006b) Hormonal and reproductive factors and risk of glioma: a prospective cohort study. Inter J Cancer 118: 1321–1324 10.1002/ijc.21467 16152609

[bib30] Navas-Acién A, Pollán M, Gustavsson P, Plato N (2002) Occupation, exposure to chemicals and risk of gliomas and meningiomas in Sweden. Am J Ind Med 42: 214–227 12210690 10.1002/ajim.10107

[bib31] Oh SW, Yoon YS, Shin SA (2005) Effects of excess weight on cancer incidences depending on cancer sites and histologic findings among men: Korea National Health Insurance Corporation study. J Clin Oncology 23: 4742–4754 10.1200/JCO.2005.11.726 16034050

[bib32] Ohgaki H, Kleihues P (2005) Epidemiology and etiology of gliomas. Acta Neuropathologica 109: 93–108 15685439 10.1007/s00401-005-0991-y

[bib33] Perry A, Louis DN, Scheithauer BW, Budka H, von Deimling A (2007) Meningeal tumours. In WHO classification of tumours of the central nervous system, Loius DN, Ohgaki H, Wiestler OD, Cavenee WK (eds), p 163. International Agency for Research on Cancer: Lyon

[bib34] Reeves GK, Pirie K, Beral V, Green J, Spencer EA, Bull D (2007) Cancer incidence and mortality in relation to body mass index in the Million Women Study: cohort study. BMJ 335: 1134–1139 17986716 10.1136/bmj.39367.495995.AE PMC2099519

[bib35] Ryan P, Lee MW, North JB, McMichael AJ (1992) Risk factors for tumors of the brain and meninges: results from the Adelaide Adult Brain Tumor Study. Int J Cancer 51: 20–27 1563840 10.1002/ijc.2910510105

[bib36] Sanson M, Cornu P (2000) Biology of meningiomas. Acta Neurochirurgica 142: 493–505 10898356 10.1007/s007010050462

[bib37] Schneider B, Pülhorn H, Röhrig B, Rainov NG (2005) Predisposing conditions and risk factors for development of symptomatic meningioma in adults. Cancer Detect Prev 29: 440–447 16188400 10.1016/j.cdp.2005.07.002

[bib38] Spencer EA, Appleby PN, Davey GK, Key TJ (2002) Validity of self-reported height and weight in 4808 EPIC-Oxford participants. Public Health Nutr 5: 561–565 12186665 10.1079/PHN2001322

[bib39] Townsend P, Phillimore P, Beattie A (1988) Health and Deprivation: Inequality and the North. Croom Helm: London

[bib40] Tulinius H, Sigfússon N, Sigvaldason H, Bjarnadóttir K, Tryggvadóttir L (1997) Risk factors for malignant diseases: a cohort study on a population of 22 946 Icelanders. Cancer Epidemiology Biomarkers and Prevention 6: 863–873 9367058

[bib41] Whitlock G, Clark T, Vander H, Rodgers A, Jackson R, Norton R, Macmahon S (2001) Random errors in the measurement of 10 cardiovascular risk factors. Eur J Epidemiol 17: 907–909 12188008 10.1023/a:1016228410194

[bib42] Wigertz A, Lönn S, Mathiesen T, Ahlbom A, Hall P, Feychting M (2006) Risk of brain tumors associated with exposure to exogenous female sex hormones. Am J Epidemiol 164: 629–636 16835295 10.1093/aje/kwj254

[bib43] World Cancer Research Fund/American Institute for Cancer Research (2007) Food, Nutrition, Physical Activity, and the Prevention of Cancer: A Global Perspective. AICR: Washington DC

[bib44] World Health Organization (1992) International Statistical Classification of Diseases and Related Health Problems, 10th revision World Health Organization: Geneva

[bib45] Zheng T, Cantor KP, Zhang Y, Chiu BCH, Lynch CF (2001) Risk of brain glioma not associated with cigarette smoking or use of other tobacco products in Iowa. Cancer Epidemiology Biomarkers and Prevention 10: 413–414 11319186

